# Study on Bottom Distributed Bragg Reflector Radius and Electric Aperture Radius on Performance Characteristics of GaN-Based Vertical-Cavity Surface-Emitting Laser

**DOI:** 10.3390/ma17133107

**Published:** 2024-06-25

**Authors:** Dominika Dąbrówka, Robert P. Sarzała

**Affiliations:** Institute of Physics, Lodz University of Technology, 217/221 Wólczańska St., 93-005 Łódź, Poland; robert.sarzala@p.lodz.pl

**Keywords:** VCSEL, GaN, DBR, electrical aperture, numerical simulation

## Abstract

This article presents the results of a numerical analysis of a nitride-based vertical-cavity surface-emitting laser (VCSEL). The analyzed laser features an upper mirror composed of a monolithic high-contrast grating (MHCG) and a dielectric bottom mirror made of SiO_2_ and Ta_2_O_5_ materials. The emitter was designed for light emission at a wavelength of 403 nm. We analyze the influence of the size of the dielectric bottom mirrors on the operation of the laser, including its power–current–voltage (LIV) characteristics. We also study the effect of changing the electrical aperture radius (active area dimensions). We demonstrate that the appropriate selection of these two parameters enables the temperature inside the laser to be reduced, lowering the laser threshold current and increasing its optical power output significantly.

## 1. Introduction

The commercial production of nitride-based vertical-cavity surface-emitting laser (VCSEL) devices presents ongoing challenges. The main difficulties are associated with the fabrication of nitride-based Distributed Bragg Reflectors (DBRs), which are essential components ensuring the proper operation of VCSELs. DBR mirrors were first demonstrated in around 1940 as alternating dielectric layers [1,2,3]. Currently, there are many materials that can be used to fabricate DBR mirrors. For VCSELs, DBR mirrors made from arsenic-based GaAs/AlGaAs materials are the most efficient due to the lattice matching of these materials. Extensive research on the epitaxial growth of AlGaAs has led to the development of an efficient manufacturing mechanism. This has enabled the mass commercial production of GaAs-based VCSELs. However, the situation is much more challenging for VCSELs based on nitride materials, which provide emissions in the visible range from violet through blue to green. Among other factors, these difficulties are caused by the specific physical properties of these materials, particularly the lattice mismatch between GaN, AlN, and InN [4]. As a result of this mismatch, native nitride DBR mirrors are difficult to produce [5,6,7]. This is one reason why, despite considerable theoretical and experimental work, nitride-based VCSELs have not yet been commercialized. Although there are laboratory-built VCSEL laser designs using nitride technology that achieve powers of several to several tens of milliwatts [5,8,9,10,11,12], many prototypes of VCSEL lasers based on GaN remain in the research phase, mainly due to the difficulty involved in manufacturing monolithic versions.

Over the years, various concepts for the realization of nitride-based VCSEL structures have emerged. These concepts can be divided for practical purposes into two groups. The first group consists of lasers with hybrid DBR mirrors, where one of the mirrors is made of semiconductor materials and the other mirror is made of dielectric materials. The second group comprises lasers, and both DBR mirrors are fabricated from dielectric materials.

Research on epitaxial nitride DBRs has been conducted since the 1990s. AlN/GaN mirrors exhibit the highest refractive index contrast (for a 403 nm wavelength, the refractive index difference is approximately 0.4). Theoretically, this allows for higher reflectivity while reducing the number of mirror pairs required [13,14]. However, the AlN/GaN combination suffers from significant lattice mismatch (~2.4%) and large differences in thermal expansion coefficients between GaN (5.59 × 10^−6^ K^−1^) and AlN (4.20 × 10^−6^ K^−1^). This can lead to cracking and consequent optical losses or even device failure [15]. These issues can be alleviated by using AlGaN and GaN materials. However, employing AlGaN/GaN layers as DBR stacks requires a large number of pairs due to the reduced refractive index contrast of the materials. Moreover, strain-related issues persist. Therefore, alternative pairs of suitable materials have been sought for use in DBR mirrors.

AlInN and GaN offer a potential solution, having reasonably matched lattice parameters. Yet, despite their relative lattice matching, several challenges arise when using these materials. One fundamental issue is the significant number of mirror pairs required to achieve high reflectivity due to the low refractive index contrast between GaN and AlInN (for a 403 nm wavelength, the refractive index difference is approximately 0.2) [16]. For instance, achieving only 98% reflectivity necessitates at least 30 pairs of such mirrors, with additional pairs required for higher reflectivity. This increases the size and cost of the devices [5]. Additionally, AlInN exhibits very low thermal conductivity (4.87 W/m∙K) [17], which complicates heat dissipation from the structure, especially if it is used in bottom DBRs. While AlInN may be suitable for use in upper emitting mirrors, the growth time for AlInN remains a problem. AlInN growth is highly temperature-sensitive. The growth of AlInN/GaN mirrors involves numerous temperature variation processes, prolonging growth times to several hours (approximately 12–14 h for 40 pairs) [18,19]. Consequently, the production of such mirrors is costly, making the prices of VECSEL devices with AlInN/GaN DBRs potentially uncompetitive on the mass market.

The most popular solution to the problems associated with the use of AlInN/GaN mirrors as bottom mirrors in VCSELs is the use of dielectric mirrors, most commonly made of SiO_2_ and Ta_2_O_5_ materials. These materials exhibit a significantly higher refractive index contrast than nitride materials (for a 403 nm wavelength, the refractive index difference is approximately 0.9). This results in high reflectivity with a much smaller number of mirror pairs (typically ranging from 5 to 15) [5]. Dielectric DBR mirrors can be employed on both sides of the laser resonator. However, due to technological challenges, a mixed approach is often used, combining dielectric mirrors (e.g., rear mirrors) with nitride mirrors (AlInN/GaN or AlN/GaN) as front mirrors in a single device. Currently, such mixed structures are the most efficient and emit the highest power [12,20,21,22,23]. Nevertheless, the fabrication of VCSELs with dielectric mirrors is significantly more challenging, as the mirrors are produced in a separate technological process. Additionally, dielectric mirrors impede the flow of heat and current. Therefore, dielectric mirrors must be used in conjunction with ITO/SiO_2_ layers and annular metallic contacts, primarily made of gold. This ensures proper current injection into the laser structure and facilitates heat dissipation from the interior towards the heat sink. Proper design of this element is crucial for the correct and efficient operation of the device.

The disadvantages associated with mirrors made of AlInN/GaN or SiO_2_/Ta_2_O_5_ have led to the development of alternative approaches to manufacturing GaN-based VCSELs. For example, Kogelnik and Li employed a cavity with one flat and one curved mirror, which together formed a stable laser resonator [24]. In 2003, Park et al. reported the operation of a GaN-based VCSEL with monolithic curved mirrors, optically pumped [25]. Over the past few years, various VCSEL structures with curved mirrors have been demonstrated [8,9,26,27,28,29,30,31,32,33]. Recently, it was demonstrated that upper DBR mirrors can be successfully replaced by high-index contrast gratings (HCG) and monolithic high-refractive-index contrast gratings (MHCG). These gratings have been applied particularly in designs for mid-infrared VCSELs with a suspended InP HCG [34,35] and near-infrared VCSELs based on GaAs integrated with monolithic HCGs made of GaAs or AlGaAs [36,37]. Similarly, it is possible to utilize HCGs made of TiO_2_ [7,38,39] and MHCGs made of GaN [40] to manufacture blue GaN-based VCSELs.

The challenges related to nitride VCSELs include not only the fabrication of DBR mirrors but also uneven current injection into the active region. This is primarily due to the low electrical conductivity of p-type GaN material. Consequently, directing current paths to ensure current penetration into the center of the structure becomes a significant design issue. The most common solution is to create an aperture in a thin dielectric layer (e.g., SiO_2_) and fill it optically with a transparent material of high electrical conductivity, such as indium tin oxide (ITO). Unfortunately, due to its high electrical conductivity, ITO also exhibits high absorption coefficients, leading to significant optical losses [41]. On the other hand, materials with lower optical losses have weaker electrical conductivity, which complicates the flow of current into the center of the structure and increases the non-uniformity of current injection into the active area. Properly designing the dimensions of the electrical aperture to allow optimal utilization of injected current into the active region to enhance the operating mode of the laser thus remains a major challenge for designers. Designing highly efficient single emitters for GaN-based VCSELs and optimizing their operational parameters would allow for the effective utilization of VCSELs in various applications, such as color displays, projectors, transparent displays, lighting devices, high-resolution printing, dense data storage and retrieval devices, visible range optical wireless communication, optical communication, LiDAR systems, medical diagnostics, biosensors, spectroscopic research, etc. [40,42,43,44,45,46]. Additionally, it would open up the possibility of constructing two-dimensional VCSEL arrays.

Given both the opportunities and challenges presented by the design of DBR mirrors for use in nitride VCSELs, hybrid designs in which monolithic GaN MHCG mirrors are employed on one side and SiO_2_/Ta_2_O_5_ mirrors are used on the other side (at the back), offer a promising compromise. Here, we present a numerical analysis of such a GaN-based VECSEL. The base structure was proposed by Hong et al. [40] and designed for light emission at a wavelength of 403 nm. The original laser operated only under pulsed conditions. Our main objective was to explore design solutions capable of improving the performance of the laser, with the particular aim of achieving continuous wave operation. First, we compared the experimental results reported by Hong et al. [40] with the results of numerical simulations to calibrate the numerical model. Then, we sought to improve the performance of the device by varying the radius of the bottom dielectric mirrors and the radius of the electrical aperture of the laser. It was demonstrated that by carefully tuning these two parameters, it is possible to reduce the temperature inside the laser, lower its threshold current, and achieve a significant increase in optical power output. 

## 2. Materials and Methods

### 2.1. Structure

**Figure 1:**
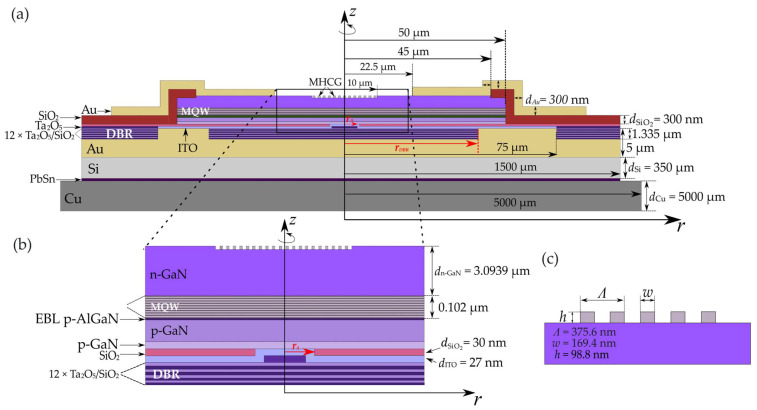
Schematic of the structure (**a**) of a nitride-based VCSEL with bottom dielectric DBR mirrors and top mirrors in the form of an MHCG; close-up (**b**) of a fragment of the VCSEL laser around the active region; schematic (**c**) of the MHCG with dimensions labeled. Figures not to scale.

**Figure 2:**
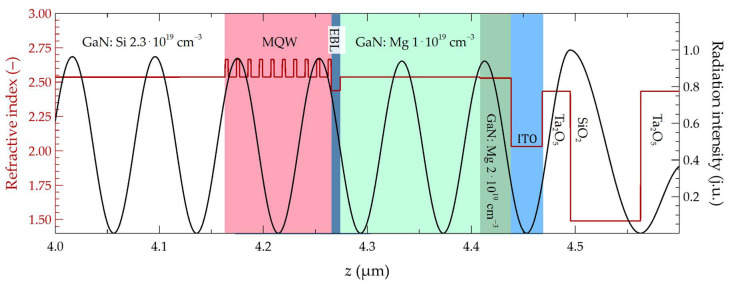
Distribution of the refractive index and standing wave in the modeled structure of a nitride-based VCSEL with a 21.5λ resonator zoomed in on key areas around the active region of the laser.

The structure of the simulated VCSEL laser was based on published works by research teams from Taiwan and Sweden [40]. The entire structure was designed for light emission at a wavelength of 403 nm. A schematic of the entire structure, including a close-up of the central part of the laser, is presented in Figure 1. The foundation of the structure consists of a silicon carrier substrate with a thickness of 350 µm. Directly on top, there is a gold layer connecting the carrier substrate to the laser structure. Next are the bottom DBR mirrors, consisting of 12 pairs of Ta_2_O_5_ and SiO_2_ layers with thicknesses of 41.4 nm and 67.1 nm, respectively, with a radius denoted as *r*_DBR_. During the simulations, this radius was varied in the range from 10 µm to 40 µm. Above the bottom DBRs is a 27 nm Ta_2_O_5_ phase-shifting layer surrounded by an ITO layer. The size of the phase-shifting layer, along with the correlated inner radius of the SiO_2_ layer, serves as the electrical aperture of the laser. The aperture has a radius of *r*_A_ and simultaneously determines the boundary of the active region of the device in the radial direction (see Figure 1b). The radius *r*_A_ is the second dimension of the laser and was varied in the range from 3.0 µm to 4.5 µm during the simulations. The active region consists of ten 3 nm In_0.1_Ga_0.9_N quantum wells separated by nine 8 nm GaN barriers. The laser resonator was designed to be 21.5 wavelengths long. The active region was designed to be positioned in two antinodes of the standing wave (see Figure 2). On the top surface of the laser is positioned an MHCG with a combined radius of 10 μm. The grating parameters are as follows: period *Λ* (375.6 nm), width *w* (169.4 nm), and height *h* (98.8 nm) (see Figure 1c). Detailed design data of the analyzed structure are provided in Table 1.

### 2.2. Numerical Model

The presented results were obtained from numerical simulations conducted using a proprietary model and computer program developed by the Photonics Team of the Institute of Physics at the Lodz University of Technology. This program is called RPSMES and enables the simulation of physical phenomena occurring, particularly during the operation of semiconductor lasers. Self-consistent calculations were performed by integrating a thermal, electrical, optical, and active region gain model. Due to the cylindrical symmetry of VCSEL lasers, calculations were performed using the cylindrical *rz* coordinate system depicted in Figure 1. This enabled the reduction of computations to 2D calculations. The main focus of the study was on the case of a laser operating in continuous wave (CW) mode. 

The temperature distribution in the laser and in the heatsink on which it rests was obtained by solving the stationary Fourier–Kirchhoff heat conduction equation:

(1) $$\nabla \left(k\left(r,z\right)\nabla T\left(r,y\right)\right)=-g\left(r,z\right),$$

where *T* is the temperature, *k* is the thermal conductivity tensor, and *g* is the volumetric heat source density. The numerical solution of the Equation (1) was obtained using the Finite Element Method (FEM). The electrical model we used is based on solving Laplace’s equation:

(2) $$\nabla \left(\sigma \left(r,z\right)\nabla V\left(r,z\right)\right)=0,$$

where *σ* is the conductivity tensor of the material and *V* is the electric potential at a point with coordinates (*r*,*z*). The Finite Element Method (FEM) was also used to solve this equation. Since recombination and generation of carriers occur in the active region, the right-hand side of Equation (2) is non-zero. Consequently, an equivalent electrical conductivity of the active region can be introduced, which was determined based on two equations. The first equation is the diode equation:

(3) $$j=j_{s}\left[exp\left(\beta U\right)-1\right],$$

where *j* is the current density, U is the voltage across the junction, and *j*_s_ and *β* are parameters characterizing the junction. The second equation, from which the distribution of current density flowing through the active region is determined, comes from the differential form of Ohm’s law:

(4) $$j=-\sigma_{z}\nabla V.$$

Based on the combination of the two dependencies, (3) and (4), the aforementioned equivalent electrical conductivity, *σ*_z_, of the active region was obtained. Since the conductivity of the junction depends on the current density flowing in a given location of the active region, which in turn depends on its conductivity, both values were iteratively determined in a self-consistent manner. The values of thermal conductivity and electrical conductivity for Au, SiO_2_, PbSn, and Cu were taken from [47,48]. For the second material forming the dielectric DBR mirror, thermal parameters were extracted from [49]. In the case of nitride materials, GaN, In_0.1_Ga_0.9_N, and Al_0.2_Ga_0.8_N, thermal parameters were taken from [50]. The parameter values (thermal and electrical conductivities of the materials in each layer of the laser) used to model the laser operation are presented in Table 1.

The determined potential distribution enables the calculation of the current density distribution throughout the laser structure. This, in turn, enables the calculation of the volumetric power density, *g*, present in Equation (1). Since temperature affects both the thermal and electrical conductivity of the individual materials constituting the laser, both Equations (1) and (2) are solved in a self-consistent mode. By determining the potential distribution throughout the device structure, it is possible to calculate the injected current density into its active region as a function of the coordinate *r*. This enables the subsequent determination of the carrier distribution in the active region by solving the diffusion equation:

(5) $$D∆n\left(r\right)-\left(A\cdot n\left(r\right)+B{\cdot n}^{2}\left(r\right)+C{\cdot n}^{3}\left(r\right)\right)+\frac{j\left(r\right)}{e\cdot d}=0\;,$$

where *D* is the ambipolar diffusion coefficient, *n* is the carrier concentration, *A* is the monomolecular recombination coefficient, *B* is the bimolecular recombination coefficient, *C* is the Auger recombination coefficient, *e* is the elementary charge, d is the thickness of the laser active region, and *j* is the current density injected into the active region as a function of the coordinate *r*.

The coefficients *A*, *B*, *C*, and *D* in Equation (5), corresponding to the active region fabricated as a multiple quantum well (MQW) InGaN/GaN, were determined based on literature data. In the literature, the values of the monomolecular recombination coefficient, *A*, for InGaN/GaN quantum wells range from 0.1 × 10^7^ s^−1^ to 11.3 × 10^7^ s^−1^ [51,52,53,54,55,56]. The most suitable relationship for this coefficient appears to be that presented in [57], which was developed based on [55]. It is expressed as

(6) $$A\left(T\right)=4\times 10^{7}\cdot {\left(\frac{T}{300\;K}\right)}^{3.8}s^{-1}.$$

As indicated by Equation (6), the value of the monomolecular recombination coefficient *A* at room temperature is 4 × 10^7^ s^−1^.

The value of the bimolecular recombination coefficient, *B*, was calculated based on the relationship presented in [57]. It was assumed to be 2.0 × 10^−11^ cm^3^s^−1^ at room temperature, according to the relationship provided based on data in [58]:

(7) $$B\left(T\right)=2.0\times 10^{-11}\cdot {\left(\frac{300\;K}{T}\right)}^{1.5}{cm}^{3}s^{-1}.$$

In the literature, the reported values of the coefficient *B* for InGaN/GaN quantum wells range from 0.15 × 10^−11^ cm^3^s^−1^ to 7.0 × 10^−11^ cm^3^s^−1^ [52,53,54,56,59].

According to the literature data, for InGaN/GaN materials, the Auger recombination coefficient, *C*, at room temperature ranges from 3.5 × 10^−34^ to 2.0 × 10^−30^ cm^6^s^−1^ [51,53,55,56,59,60]. In reference [57], a simple Formula (8) for the value of coefficient *C* based on experimental data [53,61] is presented. This formula can be used to calculate the Auger recombination coefficient for photon energies *E* corresponding to the emitted wavelength:

(8) $$C\left(E\right)=1.1\times 10^{-28}\cdot {\left(\frac{E}{1\;eV}\right)}^{-4}{cm}^{6}s^{-1}.$$

Since the simulated laser is designed for a wavelength of 403 nm, its photon energy *E* is 3.0765 eV. Upon substitution into Equation (8), this yields an Auger recombination coefficient *C* equal to 1.227 × 10^−30^ cm^6^s^−1^.

The coefficient of ambipolar diffusion, *D*, was determined based on an approximate formula describing its dependence on temperature, which was derived from experimental data [51]:

(9) $$D\left(T\right)=2.0\cdot \left(\frac{300\;K}{T}\right){cm}^{2}s^{-1}.$$

Therefore, it was assumed that the coefficient *D* at 300 K is 2.0 cm^2^s^−1^ [62,63,64,65].

Knowing the carrier distribution in the active region enables the optical gain distribution of the material within this region to be found. The optical gain is determined using Fermi’s golden rule [66]. In this case, for a single quantum well, the gain as a function of energy ℏω, in the approximation of parabolic bands, can be described by the following equation:

(10) $$g\left(\hbar \omega \right)=\sum_{m}\int_{-\infty }^{+\infty }g_{m}\left(\varepsilon \Lambda \left(\hbar \omega -\varepsilon \right)\right)d\varepsilon ,$$

where Λ is a function describing the spectral broadening, and summation is performed over all pairs of states, *m*. 

To determine the optical field distribution and the wavelength of individual modes, an optical model based on the Effective Frequency Method (EFM) was employed. This method assumes that the optical field of the eigenmodes exponentially depends on time, according to the following equation [67]:

(11) $$E\left(r,\omega \right)=E\left(r,\omega \right)exp\left(i\omega t\right).$$

The modal frequency, *ω*, from Equation (11) is an eigenvalue of the time-independent scalar wave equation with the following form [68]:

(12) $$\left[∆+\frac{\omega^{2}}{c^{2}}\varepsilon \left(r,\omega \right)\right]=E\left(r,\omega \right)=0,$$

where *ε* is the dielectric constant of the layer. The imaginary part of the complex modal frequency accounts for absorption and amplification within the resonator and the DBR mirror of the laser. After appropriate transformations and substitutions, formulas describing the absorption coefficient for modes and the wavelength of emitted radiation can be obtained. Using the EFM method in conjunction with the thermal and electrical model, we can determine the laser threshold for specific modes and the distribution of the electromagnetic field intensity of a given mode perpendicular and parallel to the resonator axis. For optical calculations, the values of the refractive index and absorption coefficient were taken from [68,69] for Au and from [70] for SiO_2_. For the second material forming the DBR mirrors, the optical parameters were taken from [71]. In the case of nitride materials, GaN, In_0.1_Ga_0.9_N, and Al_0.2_Ga_0.8_N, the optical parameters were taken from [50]. The set of optical parameters (refractive indices and absorption coefficients) used in the calculations is provided in Table 2.

Both the threshold parameters of the laser and its *LIV* characteristics were determined. The output power of the laser light *P*_out_(*I*) was calculated using the equation

(13) $$P_{out}\left(I\right)=\eta_{i}\left(\frac{\alpha_{m}}{\alpha_{m}+\alpha_{i}}\right)\frac{h\nu }{e}\left(I-I_{th}\right),$$

where *η*_i_ is the internal quantum efficiency (*η*_i_ = *η*_inj_ × *η*_prom_), consisting of the injection efficiency into the wells (*η*_inj_) and the radiative recombination efficiency (*η*_prom_), *α*_m_ represents the modal edge losses due to light emission, *α*_i_ is the total internal modal losses, hν is the photon energy, *e* is the elementary charge, *I* is the current flowing through the laser, and *I*_th_ is the threshold current of the laser associated with the temperature of its active region [49]. In our model, the threshold current is determined for different temperatures of the heat sink. The appropriate value is then determined by approximation for the currently prevailing thermal conditions in the laser. In the case of the studied VCSEL structure, it was assumed that the internal quantum efficiency is 84% because the efficiency of injection into the active region was estimated to be about 84%. Values for the efficiency of individual nitride-based VCSEL lasers are difficult to find in the literature. For InGaN-based laser diodes, injection efficiency values range from 100% to 75%, depending on factors such as the concentration of magnesium doping and the thickness of the electron-blocking layer (EBL) [72,73]. The radiative recombination efficiency was assumed to be 100% [73,74,75].

All the models described above are based on the mutual interaction between thermal, electrical, gain, and optical phenomena. They operate in a self-consistent mode, meaning that each model takes into account the interactions between different phenomena occurring in the laser. Detailed descriptions of the models used for the calculations can be found in [76,77,78,79], while descriptions of the integration of these models for VCSEL laser calculations can be found in [77,78,79].

**Figure 3:**
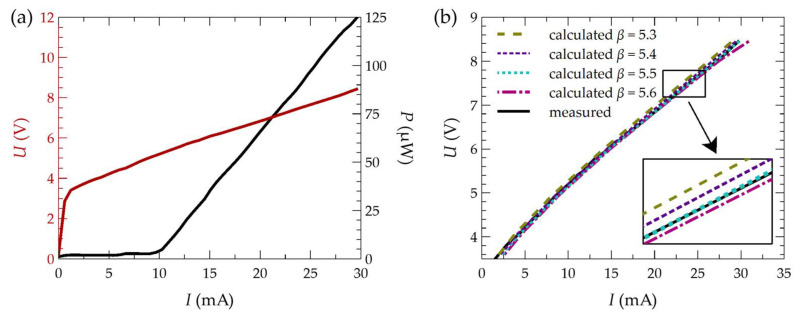
Measured LIV characteristic of the VCSEL laser with monolithic HCG mirrors in pulsed operation conditions (**a**) [40] and current–voltage characteristics (**b**) obtained for different values of the junction parameter, including an approximate section.

One of the first steps in fitting the model to experimental results was to determine the parameter *β* characterizing the active region of the laser. This parameter, associated with the diode Equation (3), was adjusted to the light–current–voltage (LIV) characteristics presented in [40] for a laser with an identical structure to the one considered in this study. This structure had an electrical aperture radius of 4.5 µm and DBR bottom mirrors with a radius of 40 µm. In further consideration, a structure with these dimensions will be referred to as the base structure. The fitting was performed by calculating the current–voltage characteristic *U(I)* for various values of the parameter *β* so that the numerically obtained characteristic matched the one obtained experimentally under pulsed operation conditions. Figure 3a shows the measured *LIV* characteristic [40]. Figure 3b presents the obtained *U(I)* characteristics using simulations for four sample values of *β*, assuming a structure temperature of 300 K. Based on the conducted simulations and comparison of the measured *U(I)* characteristic with the calculated characteristics for different values of *β* (see Figure 3b), it was found that the best-matched characteristic was obtained for a value of *β* equal to 5.5 $V^{-1}.$ By employing the thermal and optical parameters described above, along with the coefficients *A*, *B*, *C*, and *D*, as well as the internal quantum efficiency, alignment was achieved between the numerically determined threshold current value for the baseline structure (*r*_A_ = 4.5 µm and *r*_DBR_ = 40.0 µm) operating under pulsed conditions and the value reported in the study [40], obtained experimentally. This value amounts to 10.2 mA. Such close agreement indicates the appropriate selection of the model parameters, which is necessary for the further computations outlined in the study.

## 3. Results and Discussion

**Figure 4:**
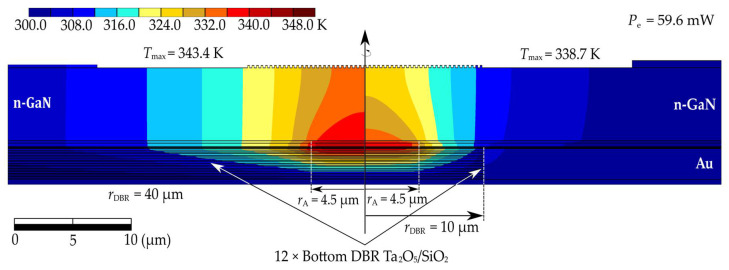
Temperature distribution for a VCSEL laser with a 21.5λ resonator, designed for an emission wavelength of 403 nm. The temperature distribution in the base structure supplied with 59.6 mW of power (left side); the temperature distribution in a structure with the bottom mirror radius reduced to 10 µm under the same electrical power supply conditions (right side).

The flow of current through the laser structure induces heat generation inside it, leading to an increase in temperature throughout the device. This temperature rise adversely affects the performance parameters and operation of the laser. The increase in temperature particularly deteriorates the material parameters, such as the thermal conductivity of individual materials. Consequently, we encounter unfavorable feedback, leading to further temperature rises. Limiting these detrimental effects is one of the primary challenges in the design of laser structures. Figure 4 (left side) shows the temperature distribution in the base structure for a current intensity of 10.2 mA, corresponding to an electrical power of 59.6 mW. For comparison, the temperature distribution in the same structure and for the same supplied electrical power, but with a reduced radius of the bottom DBR mirrors (from 40 µm to 10 µm), is shown in Figure 4 (right side). During the calculations, it was assumed that the temperature at the bottom of the heat sink was 300 K. Based on the generated temperature distributions (see Figure 4), it can be observed that the change in the radius of the bottom dielectric mirrors significantly influences the maximum temperature change in the structure. Hence, the dielectric mirrors (Ta_2_O_5_/SiO_2_) significantly affect the temperature within the device, and also affect the laser performance parameters. This is because these mirrors have low thermal conductivity (0.45 W/(m∙K) and 1.38 W/(m∙K) for Ta_2_O_5_ and SiO_2_, respectively) compared to materials such as (Al)GaN, making heat dissipation from devices operating in CW mode challenging. In the analyzed structure, the mirrors are surrounded by an embedded gold ring contact. Gold exhibits high thermal conductivity (317.1 W/(m∙K)), effectively aiding in heat dissipation from the interior of the structure towards the heat sink. Consequently, reducing the radius of the bottom DBR mirrors in the analyzed structure improves the device’s thermal properties. Reducing the mirror radius from 40 µm to 10 µm resulted in a reduction in temperature increase of nearly 11%. The thick n-type GaN layer located in the laser resonator plays a significant role in this process. In the case of narrowed DBR mirrors, this layer facilitates more two-dimensional heat dissipation from the active region.

**Figure 5:**
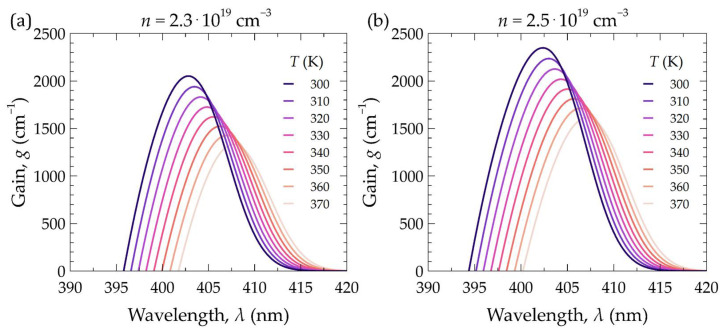
Dependence of the gain in the laser’s active region on wavelength for various temperatures and two different carrier concentrations: (**a**) 2.3 × 10^19^ cm*^−^*^3^ and (**b**) 2.5 × 10^19^ cm*^−^*^3^.

As mentioned previously, temperature affects the material parameters, particularly relating to changes in the bandgap of the materials forming the active region of the laser and the occupation of states by the carriers. Such changes result in the shifting of the gain spectrum of the active region and changes in its value. In Figure 5, calculated distributions of the material gain of the laser’s active region are presented as a function of wavelength for two carrier concentration values in the active region. The plots were generated for various temperatures of the active region (from 300 K to 370 K). For example, at a temperature of 300 K, the maximum gain value for a concentration of 2.3 × 10^19^ cm*^−^*^3^ is 2050 cm*^−^*^1^ for a wavelength of 403.0 nm (see Figure 5a). An increase in temperature from 300 K to 310 K results in a decrease in gain from 2050 cm*^−^*^1^ to 1939 cm*^−^*^1^ and a shift of the gain spectrum maximum from 403.0 nm to 403.5 nm, i.e., d*λ*/d*T* = 0.05 nm/K. Larger temperature increases are associated with even greater decreases in gain. A temperature increase of 50 K leads to a gain reduction of approximately 25%. Changing the carrier concentration from 2.3 × 10^19^ cm*^−^*^3^ to 2.5 × 10^19^ cm*^−^*^3^ increases the gain by approximately 300 cm^−1^. Thus, excessive temperature rises inside the laser can significantly affect the laser threshold current, limiting the optical power emitted by the laser or even rendering its operation impossible. Therefore, ensuring the proper design of the laser structure in terms of heat dissipation (especially through the appropriate design of the bottom DBR mirrors) becomes extremely important.

**Figure 6:**
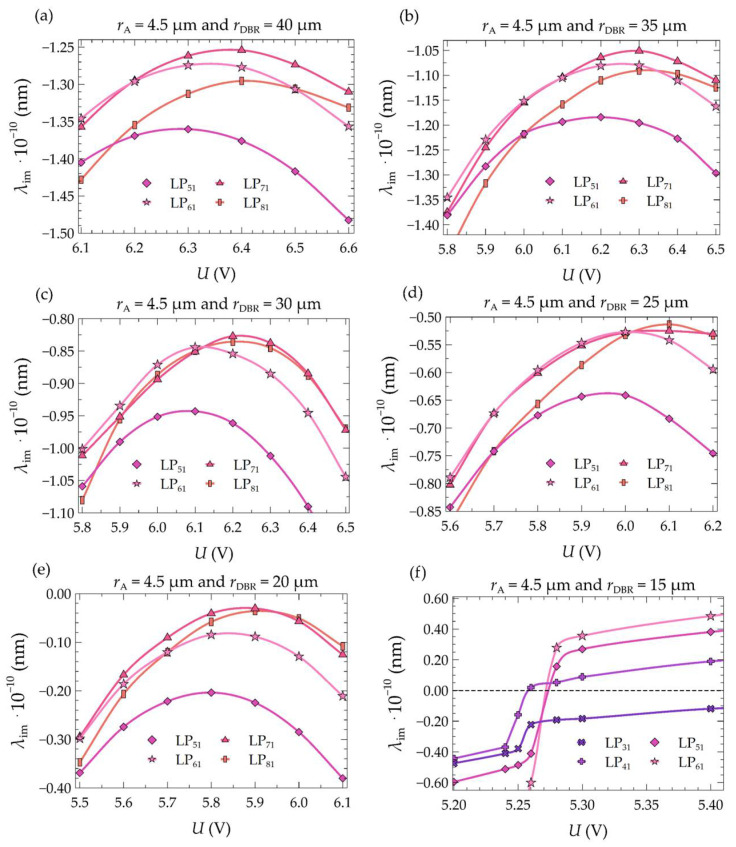
Dependence of optical losses on the supply voltage for selected modes in lasers with an electrical aperture radius of 4.5 µm and various radii of the bottom DBR mirrors: (**a**) 40 µm, (**b**) 35 µm, (**c**) 30 µm, (**d**) 25 µm, (**e**) 20 µm, and (**f**) 15 µm.

The laser structure presented in [40], which was chosen as the baseline structure in this study, operated only under pulsed conditions. According to our results, this was due to excessive temperature rises within its interior. To illustrate the excessive temperature rise, a numerical analysis of the operation of the base structure in continuous wave (CW) mode was conducted. Optical losses of excited modes were calculated. The baseline structure had an electrical aperture with a radius of 4.5 µm and bottom dielectric DBR mirrors with a radius of 40 µm. The optical losses for selected modes, depending on the supply voltage, are shown in Figure 6. In the case of the baseline structure, the strongest modes in the structure are modes LP_61_, LP_71_, and LP_81_ (see Figure 6a) for the investigated voltage ranges (6.1–6.6 V). It can be inferred from Figure 6a that even when achieving the smallest losses (i.e., for U = 6.4 V), the strongest mode does not have a sufficiently high optical gain to reach a positive value of λ_im_, resulting in failure to achieve the laser threshold even for this strongest mode. The excitation conditions of individual modes in the active region of the laser can be improved by lowering its temperature—for example, by reducing the radius of the bottom DBR mirrors. Subsequent plots in Figure 6, from Figure 6a–e, show mode losses with decreasing radii of the bottom mirrors by increments of 5 µm. As can be seen, reducing the radius of the bottom DBR mirrors positively affects the modal gain of individual modes. Unfortunately, even reducing this radius to 20 µm (Figure 6e) does not allow for the achievement of the laser threshold. As shown in Figure 6f, for a laser with an electrical aperture of the active region of 4.5 µm, it is possible to achieve continuous wave operation—for example, in a structure with bottom DBR mirrors with a radius of 15 µm. However, the laser operating conditions can be further improved by optimizing the size of the active region.

The electrical aperture of a laser with a radius *r*_A_, which simultaneously determines the boundary of the active region of the device radially, is defined by the inner radius of the SiO_2_ ring layer (see Figure 1b). The mode on which the laser operates and how the mode distribution matches the current distribution depends on the radius of the active region. In further numerical simulations, two parameters were combined: the radius of the active region (*r*_A_) and the radius of the bottom DBR mirrors (*r*_DBR_). Various configurations of these parameters were analyzed to understand their impact on temperature, laser threshold current, and the possibility of achieving continuous wave operation.

**Figure 7:**
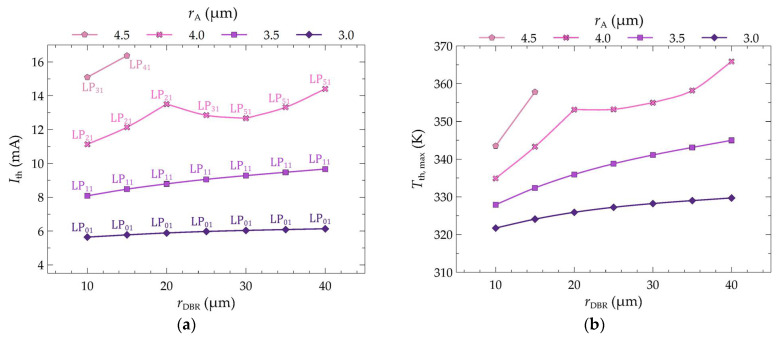
Dependence of (**a**) threshold current (*I*_th_) and (**b**) maximum temperature *T*_th,max_ on the radius (*r*_DBR_) of bottom DBR mirrors and the radius *r*_A_ of the active region. The numbers on the *I*_th_(*r*_DBR_) graph indicate the threshold mode number corresponding to the marked threshold current value.

In the simulations, seven values of the bottom DBR mirror radius were considered in the range from 10 to 40 µm with a step of 5 µm, along with four values of the aperture radius: 3.0, 3.5, 4.0, and 4.5 µm. Based on the calculations, graphs were plotted to show the dependence of the threshold current (*I*_th_) (see Figure 7a) and the corresponding maximum temperature in the active region (*T*_th,max_) (Figure 7b) on the radius (*r*_DBR_) of the bottom DBR mirrors. The graphs were plotted for different values of the aperture radius (*r*_A_) of the active region. The precise values of the obtained threshold currents are presented in Table 3.

As mentioned above, for five values of the mirror radius with an aperture radius of 4.5 µm, it is impossible to achieve the laser threshold in continuous wave mode. This is due to the rapid increase in temperature inside the structure with an increase in *r*_DBR_ for larger values of *r*_A_, as shown in Figure 7b. However, reducing the aperture radius enables the laser threshold to be achieved even for the largest considered values of the mirror radius. Additionally, in lasers with smaller electrical apertures, slight changes in the threshold current are observed with changes in the mirror radius. The changes in current are usually in the range of 0.05–0.39 mA, whereas for larger aperture values, the changes are in the range of 0.60–1.37 mA. For lasers with aperture radii of 4.0 µm and 4.5 µm, no continuous dependence of the threshold current on the mirror radius is observed. This is because these lasers operate on higher-order modes, the order of which depends additionally on the mirror radius (via its influence on the temperature distribution in the active region and thus on the material gain of this region). For an aperture radius of 4.0 µm, a decrease in the threshold current value is observed when the mode order switches to a higher mode order. Within the examined range, two such decreases are observed: when changing the mirror radius from 20 µm to 25 µm, the LP_21_ mode switches to LP_31_, and then for the change from 25 µm to 30 µm LP_31_ switches to LP_51_.

In summary, optimizing the size of the electrical aperture by changing its radius reduces the threshold current values, resulting in a decrease in the maximum temperature in the active region at the laser threshold. Increasing the aperture radius of the active region increases the difference between the maximum temperatures at the threshold for lasers with mirror radii of 10 µm and 40 µm (see Figure 7b). These differences are 7.9 K, 17.1 K, and 30.9 K for aperture radii of 3.0 µm, 3.5 µm, and 4.0 µm, respectively.

**Figure 8:**
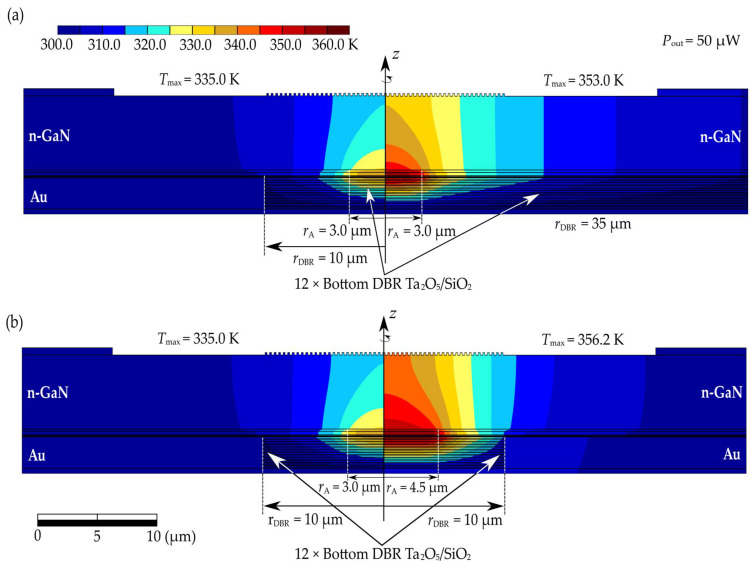
Temperature distribution for a VCSEL laser with a 21.5λ resonator and dielectric mirrors, designed for emission of a 403 nm wavelength. The laser output power is 50 µW. Distribution (**a**) corresponds to a structure with an aperture radius of 3.0 µm and bottom mirrors with radii of 10 µm (left part of the figure) and 35 µm (right part of the figure). Distribution (**b**) corresponds to a structure with bottom mirrors with a radius of 10 µm and an electrical aperture radius of 3.0 µm (left part of the figure) or 4.5 µm (right part of the figure).

From the results presented so far, it is evident that by improving the thermal design of the laser, we can achieve continuous wave operation. Figure 8 clearly illustrates how the thermal conditions inside the laser change depending on the radius of its bottom DBR mirrors and the radius of its electrical aperture. In Figure 8, temperature distributions are compared for lasers with an output power of 50 µW, different electrical aperture radii, and different bottom DBR mirror radii. Comparing the thermal conditions within the laser at the same laser output power, we can observe the beneficial effects of changing both radii. For the laser with an active region radius of 3.00 µm and laser output power of 50 µW, reducing the radius of the DBR mirrors (from 35 µm to 10 µm) results in a decrease in the maximum temperature rise inside the laser by 18.0 K, which is almost 34% (see Figure 8a). Similarly, in the case of mirrors with a radius of 10 µm, reducing the electrical aperture radius (from 4.5 µm to 3.0 µm) decreases the maximum temperature rise by 21.2 K, which is almost 38% (see Figure 8b).

**Figure 9:**
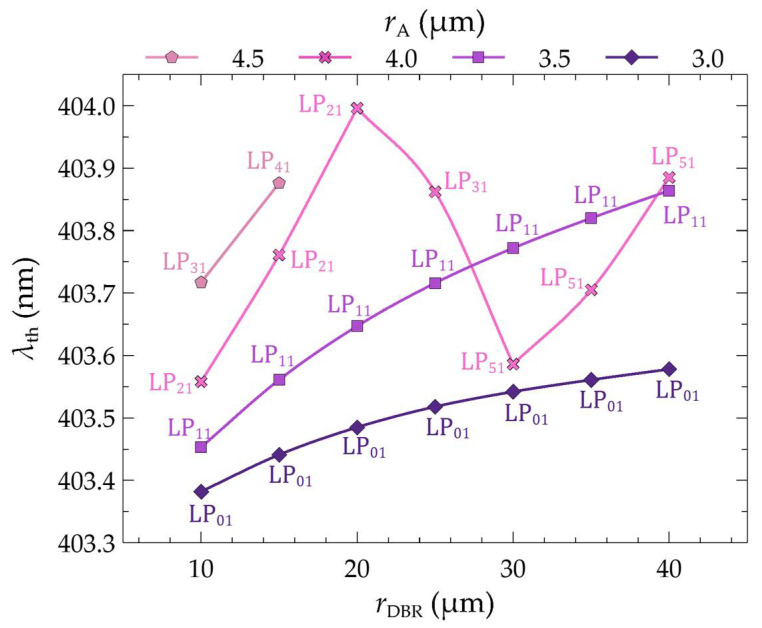
Wavelengths of threshold modes of an MHCG GaN VCSEL laser as a function of the radius of the bottom DBR mirrors (*r*_DBR_) and the aperture radius (*r*_A_). The points on the plot denote the mode numbers corresponding to the marked wavelengths.

**Figure 10:**
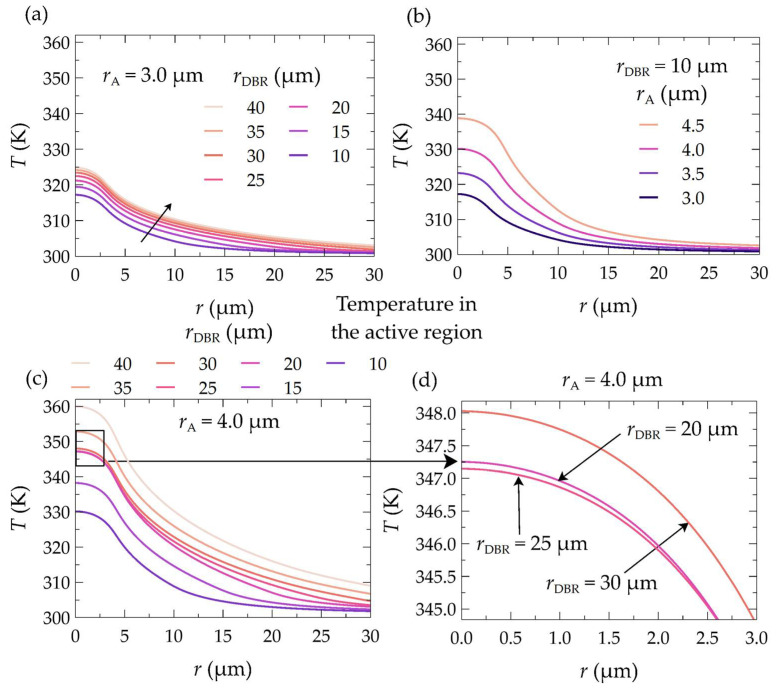
Temperature distribution in the active region of the laser along the horizontal axis in the plane of the active region, obtained for (**a**) an electrical aperture radius of 3.0 µm and various bottom DBR mirror radii, (**b**) a bottom mirror radius of 10 µm and four different electrical aperture sizes, and (**c**) an electrical aperture radius of 4.0 µm and various bottom DBR mirror radii. In plot (**c**), a rectangle marks the section of the plot presented in plot (**d**).

The significant temperature changes within the laser cavity presented above result from improved heat dissipation from the interior towards the heat sink. The temperature changes also result from the better matching of the lasing mode distribution to the injected current distribution into the active region and, as a consequence, from better matching to the gain distribution. The relationship between the wavelength of the threshold modes and their order as a function of the radius of the bottom DBR mirrors is shown in Figure 9. As can be seen, as the size of the electrical aperture increases, the mode order in which the laser operates also increases. An aperture with a radius of 3.0 µm ensures laser operation on the lowest mode, i.e., LP_01_. Increasing the aperture size also increases the order of the threshold mode. Hence, for *r*_A_ equal to 3.5 µm, the threshold modes are LP_11_. For both aperture radii of the active region, 3.0 µm and 3.5 µm, a change in the radius of the bottom DBR mirrors (within the considered range of changes) does not result in a change in the order of the threshold mode. In this case, an increase in the radius of the DBR mirrors only leads to an increase in the wavelength of a given mode. This is due to the increase in the temperature within the structure (see Figure 10a,b), which in turn leads to an increase in the refractive index and, consequently, to the optical length of the resonator.

The situation is different for larger electrical apertures. In such cases, changes in the order of the threshold mode are observed with an increase in the radius of the bottom DBR mirrors. For an aperture radius of 4.0 µm and mirror radii ranging from 10 µm to 20 µm, the laser operates on mode LP_21_. Further changes in *r*_DBR_ result in the laser operating on threshold modes of higher orders. For *r*_DBR_ = 25 µm, it is mode LP_31_, and for values in the range of 30–40 µm, the laser operates up to mode LP_51_. Similar transitions are also observed in the case of an aperture radius of 4.5 µm. Although the general trend is an increase in the mode wavelength with an increase in the radius of the DBR mirrors, in some instances, we observe a change in the wavelength value from higher to lower. This occurs, for example, for a laser with an aperture radius of 4.0 µm and a change in the radius of the DBR mirrors from *r*_DBR_ = 20 µm to *r*_DBR_ = 25 µm. This change in the wavelength from higher to lower is due to a decrease in the temperature within the structure (see Figure 10c,d). The decrease in temperature results from more favorable excitation conditions for higher-order modes (*r*_DBR_ = 25 µm).

**Figure 11:**
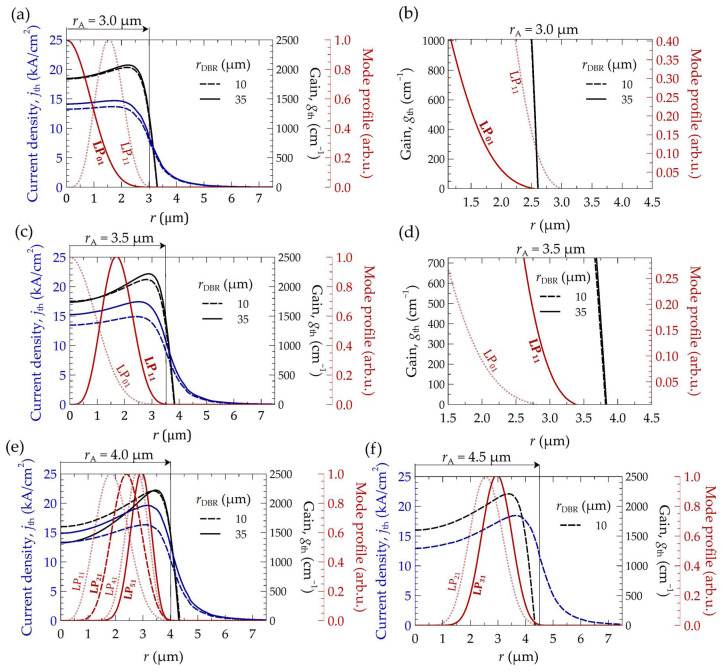
Profiles of the threshold modes (solid red line) and adjacent modes (dotted red lines), the distributions of injected threshold current density *j*_th_ into the active region of the laser (blue curves), and the gain within the active region (black curves), as functions of two radii, *r*_DBR_, of the bottom DBR mirrors: 10 µm (dashed lines) and 35 µm (solid lines), and as functions of four values of the aperture radius *r*_A_: (**a**) 3.0 µm; (**b**) 3.0 µm—an enlargement of the significant portion of gain in the active region and mode profiles; (**c**) 3.5 µm; (**d**) 3.5 µm—an enlargement of the significant portion of gain in the active region and mode profiles; (**e**) 4.0 µm; (**f**) 4.5 µm—in this case, no results are shown for mirrors with a radius of 35 µm due to the lack of lasing in continuous-wave mode.

Figure 11 presents the distributions of threshold current density, *j*_th_, injected into the active region as a function of two radii, *r*_DBR_, of the bottom DBR mirrors (10 µm (dashed line) and 35 µm (solid line)) and four values of the aperture radius, *r*_A_. The current density distributions correspond to the curves plotted in blue. As shown in Figure 11, for small active region apertures (3.0 µm), changes in the radius of the DBR mirrors cause relatively small variations in the injected current density distribution within the active region. Changes in the values and distribution of the injected threshold current become more pronounced with increasing electrical aperture sizes and increasing mirror radii. The current density distribution becomes more non-uniform, resulting in a larger amount of current flowing at the aperture edge. In VCSELs with larger active areas, we observe the current-crowding effect near the active region edge, leading to the excitation of higher-order transverse modes. The increase in laser threshold currents further exacerbates the rise in temperature (see Figure 7), leading to higher current density values being needed to achieve lasing action (see Figure 11e,f). Thus, a self-propagating negative effect occurs. The current-crowding effect intensifies (compare, for example, Figure 11e,f), leading to further temperature increase in the laser, operation on higher modes, spectrum broadening, and optical gain reduction. All of these factors influence the laser’s light–current–voltage (LIV) characteristics.

In Figure 11, we can observe why the laser with an aperture radius of 3.0 μm operates on mode LP_01_ rather than LP_11_, which appears to have better overlap with the optical gain distribution at first glance. The zoomed-in area of plot 11a (see Figure 11b) reveals that above a certain point (above *r* = 2.6 μm), mode LP_11_ incurs greater losses than mode LP_01_ because the gain for this mode becomes negative beyond this *r* value. Conversely, for a slightly larger aperture (*r*_A_ = 3.5 μm), the laser operates on mode LP_11_. This mode exhibits significantly better overlap with the gain distribution, as depicted in Figure 11c,d.

**Figure 12:**
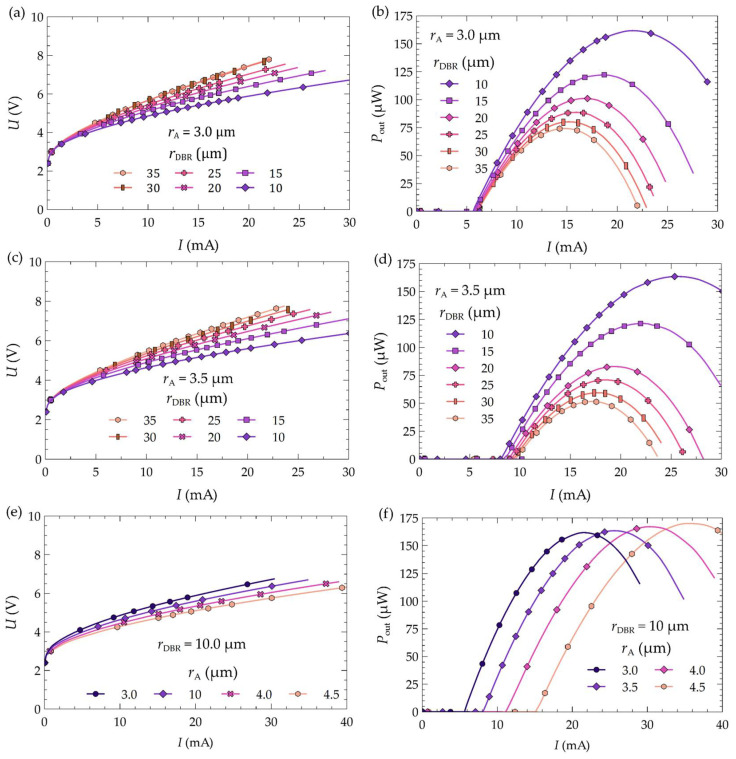
Current–voltage characteristics for two different values of the aperture radius: (**a**) *r*_A_ = 3.0µm and (**c**) 3.5 µm, at various values of the DBR mirror radius, and (**e**) for the lowest considered value of the DBR mirrors radius *r*_DBR_ = 10 µm at various dimensions of the electrical aperture. Power–current characteristics for the considered values of the aperture radius: (**b**) *r*_A_ = 3.0 µm and (**d**) 3.5 µm at various values of the dielectric mirror radius, and (**f**) for the lowest considered value of the DBR mirrors radius *r*_DBR_ = 10 µm at various dimensions of the electrical aperture of the laser.

Figure 12 illustrates the characteristics of the studied laser as a function of the laser drive current for different values of the aperture radius *r*_A_ of its active region and for various values of the radius of the bottom DBR mirrors *r*_DBR_ for which CW operation is possible. Figure 12a,b show the characteristics for *r*_A_ = 3.0 μm as a function of the radius of the bottom mirrors, ranging from 10 μm to 35 μm. The characteristics for a slightly larger aperture, i.e., *r*_A_ = 3.5 μm, are presented in Figure 12c,d. Figure 12e,f compare the LIV characteristics for different values of the electrical aperture radius with the same radius of the bottom DBR mirrors (*r*_DBR_ = 10 μm). As mentioned previously, by changing the radius of the mirrors and the aperture radius of the laser’s active region, we can influence the temperature within the laser and optimize the utilization of the injected current into its active region, thus improving the laser’s operating conditions. Based on the current–voltage characteristics presented in Figure 12, we can also observe that increasing the radius of the bottom dielectric mirrors shifts these characteristics upwards, which is related to the increase in the electrical resistance of the structure (elongation of the current paths). A similar but smaller effect occurs when the radius of the electrical aperture of the laser is reduced (see Figure 12e).

**Figure 13:**
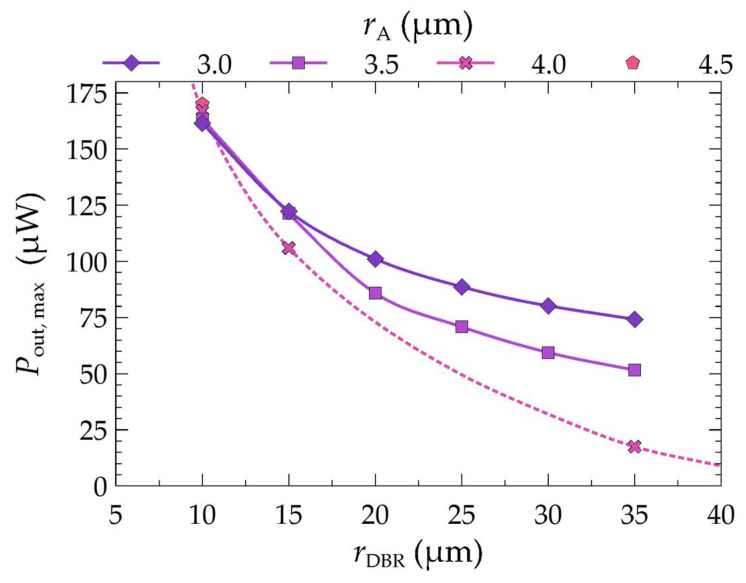
Maximum powers obtained for different aperture radii *r*_A_ of the active region of the laser as a function of the radius of its bottom DBR mirrors *r*_DBR_.

By selecting the appropriate combination of the bottom DBR mirror radius and the active region aperture radius, it is possible to not only reduce the temperature inside the laser and its threshold current but, most importantly, increase the maximum optical power emitted by the device. From the power–current characteristics shown in Figure 12b,d, we can observe that changes in the maximum powers in the laser are closely related to the change in the radius of its bottom DBR mirrors. Based on the LI characteristics from Figure 12b,d,f, Figure 13 shows the maximum optical powers emitted by the laser as a function of its active region aperture radius *r*_A_ and the radius of its bottom DBR mirrors *r*_DBR_. The simulations show that reducing the radius of the bottom mirrors not only leads to changes in the threshold current (lowering its value) or the mode order on which the laser operates (see Figure 7), but also increases the laser’s maximum optical power. For example, in the case of a laser with an electrical aperture radius of 3.0 μm, changing the radius of the DBR mirrors from 35 μm to 10 μm reduces the threshold current by 0.45 mA (see Table 3), while doubling the maximum laser power (see Figure 12b or Figure 13). Larger apertures exhibit even greater changes in both the threshold current and the maximum power. For instance, for an aperture radius of 4.0 μm the threshold current is reduced by 2.19 mA (see Table 3), and the maximum power is increased by a factor of 9 from 17.5 μW to 167.1 μW (see Figure 13).

A slightly different situation is observed for cases with bottom mirrors of radius 10 μm (see Figure 12f). In these cases, the maximum laser power is achieved for an aperture of 4.5 μm (170.2 μW) and the lowest for 3.0 μm (161.5 μW). Increasing the radius of the bottom DBR mirrors results in the lowest powers being obtained for an aperture of 4.5 μm and the highest for 3.0 μm (see Figure 13). In the case of DBR mirrors with a radius *r*_DBR_ = 10 μm, the thermal conditions inside the laser are much better than for mirrors with a larger radius. Under these conditions, the volume of the active region plays a significant role in the laser’s emitted optical power. A larger active region volume leads to an increase in the laser’s power, and distributing a similar amount of generated heat over a larger area contributes to lowering the temperature inside the laser. This effect can be seen in Figure 12f, which compares the LI characteristics of lasers with a bottom DBR mirror radius of 10 μm.

## 4. Conclusions

This study has presented the results of a numerical analysis of a VCSEL laser designed to emit light at a wavelength of 403 nm. The laser’s initial structure was adopted from [40]. It was constructed using nitride materials in a hybrid design. The bottom (rear) mirrors were made as dielectric DBR mirrors, while the top mirrors were formed as monolithic high-contrast grating (MHCG) subwavelength gratings. In its original version, the laser had dielectric DBR mirrors with a radius of 40 µm and an electric aperture radius of 4.5 µm. The laser operated only in pulse mode. Comparing our numerical results with experimental data from reference [40] enabled the calibration of a numerical model. Simulations performed using the numerical model demonstrate that appropriate selection of the size of the bottom DBR mirrors and the electric aperture of the laser can improve the thermal conditions inside the device. Changing the *r*_DBR_ from 40 µm to 10 µm and *r*_A_ from 4.5 µm to 3.0 µm enables the temperature rise inside the laser during operation to be reduced by nearly 40%. As a result, the threshold parameters of the laser are improved. For example, by reducing the electric aperture radius of the laser by 1.5 µm (from 4.5 µm to 3.0 µm, with the *r*_DBR_ reduced to 10 µm), we can decrease the laser’s threshold current by almost 2.6 times. Consequently, continuous-wave operation becomes feasible, and proper adjustment of the size of the bottom DBR mirrors, and the electric aperture radius significantly increases the optical power emitted by the laser. For instance, in the case of a laser with an electric aperture radius of 3.0 μm, changing the *r*_DBR_ from 35 μm to 10 μm can double the maximum power of the laser. For larger apertures, even greater changes in threshold current and maximum optical power are achievable. For an aperture radius of 4.0 μm, a similar change in mirror size leads to a reduction in the laser’s threshold current by nearly 16.5%, while its maximum emitted optical power increases up to nine times.

In summary, based on the conducted numerical analysis of the presented nitride VCSEL laser structure, it can be concluded that proper tuning of the size of the active area and the size of the bottom DBR mirrors can enable operation in continuous-wave mode, reduce the threshold current, and significantly increase the emitted optical power.

## Figures and Tables

**Table 1:** Design data of the modeled VCSEL lasers and thermal parameters of materials used in simulations: *k*_300K_—thermal conductivity at 300 K; *σ*_300K_—electrical conductivity at 300 K; *k*_400K_—thermal conductivity at 400 K; *σ*_400K_—electrical conductivity at 400 K; QW—quantum well; B—barrier; EBL—electron-blocking layer.

Layer		Material		Thickness (μm)	Doping (cm^−3^)	*k*_300K_ (W/(m·K))	*k*_400K_ (W/(m·K))	*σ*_300K_ (S/m)	*σ*_400K_ (S/m)
Contact on the n-side		Au		0.3	–	317.10	310.70	4.4 × 10^7^	3.2 × 10^7^
Insulator		SiO_2_		0.3	–	1.38	1.38	1.0 × 10^−6^	1.0 × 10^−6^
MHCG		u-GaN		0.0988	–	72.19	48.26	5.2 × 10^4^	4.9 × 10^4^
Spacer on the n-side		n-GaN		3.0939	Si: 2.3 × 10^19^	82.61	55.22	5.2 × 10^4^	4.9 × 10^4^
Active region	10 × QW	n-In_0.1_Ga_0.9_N		0.003	Si: 0.1 × 10^19^	7.15	4.78	263.26	249.69
	9 × B	GaN		0.008	undoped	59.73	39.93	256.35	242.02
EBL		p-Al_0.2_Ga_0.8_N		0.0088	Mg: 2.2 × 10^19^	14.65	19.38	18.54	75.79
Spacer on the p-side		p-GaN		0.1343	Mg: 1.0 × 10^19^	83.35	55.72	71.27	243.76
		p-GaN		0.03	Mg: 2.0 × 10^19^	64.94	43.41	102.42	350.30
Aperture		SiO_2_		0.03	–	1.38	1.38	1.0 × 10^−6^	1.0 × 10^−6^
		ITO		0.030	–	3.20	3.20	1.0 × 10^6^	1.0 × 10^6^
Phasing layer		Ta_2_O_5_		0.027	–	0.45	0.45	1.0 × 10^−7^	1.0 × 10^−7^
Bottom DBR mirrors		12×	Ta_2_O_5_	0.0414	–	0.45	0.45	1.0 × 10^−7^	1.0 × 10^−7^
			SiO_2_	0.0676	–	1.38	1.38	1.0 × 10^−6^	1.0 × 10^−6^
Contact on the p-side		Au		5.0	–	317.10	317.10	4.4 × 10^7^	3.2 × 10^7^
Carrier substrate		Si		350.0	–	150.00	150.0	1.0 × 10^5^	1.0 × 10^5^
Solder		PbSn		10.0	–	50.00	50.50	6.7 × 10^6^	6.7 × 10^6^
Heat sink		Cu		5000	–	400.80	392.47	5.8 × 10^7^	4.2 × 10^7^

**Table 2:** Optical parameters of materials used in simulations: *n*_r,300K_—refractive index at 300 K; *α*_300K_—absorption coefficient at 300 K; *n*_r,400K_—refractive index at 400 K; *α*_400K_—absorption coefficient at 400 K; QW—quantum well; B—barrier; EBL—electron-blocking layer.

Layer		Material		Doping (cm^−3^)	*n*_r,300K_ (–)	*n*_r,400K_ (–)	*α*_300K_ (1/cm)	*α*_400K_ (1/cm)
Contact on the n-side		Au		–	5.3935	5.3935	1.8 × 10^6^	1.8 × 10^6^
Insulator		SiO_2_		–	1.4893	1.4893	0.02	0.02
MHCG		u-GaN		–	2.5137	2.5314	18.61	23.38
Spacer on the n-side		n-GaN		Si: 2.3 × 10^19^	2.5137	2.5314	18.61	23.38
Active region	10 × QW	n-In_0.1_Ga_0.9_N		Si: 0.1 × 10^19^	2.6574	2.6768	Gain ^1^	Gain ^1^
	9 × B	GaN		undoped	2.5320	2.5497		
EBL		p-Al_0.2_Ga_0.8_N		Mg: 2.2 × 10^19^	2.4332	2.4478	0.16685	0.2988
Spacer on the p-side		p-GaN		Mg: 1.0 × 10^19^	2.5308	2.5485	6.208	11.237
		p-GaN		Mg: 2.0 × 10^19^	2.5295	2.5472	14.085	24.1258
Aperture		SiO_2_		–	1.4893	1.4893	0.02	0.02
		ITO		–	2.0311	2.0311	3257.81	3257.81
Phasing layer		Ta_2_O_5_		–	2.4337	2.4337	97.635	97.635
Bottom DBR mirrors		12 ×	Ta_2_O_5_	–	2.4337	2.4337	97.635	97.635
			SiO_2_	–	1.4893	1.4893	0.02	0.02
Contact on the p-side		Au		–	5.3935	5.3935	1.8 × 10^6^	1.8 × 10^6^

^1^ The value obtained using the gain model described by Equation (10).

**Table 3:** Threshold current values for lasers with different electrical aperture radii and different bottom DBR mirror radii for continuous wave operation. Structures that do not operate in continuous wave mode are designated as “not working”.

CW	*I*_th_ (mA)			
$r_{DBR}\;[\mu m]$	$r_{A}\;[\mu m]$			
	3.0	3.5	4.0	4.5
10	5.64	8.09	11.13	15.09
15	5.77	8.48	12.14	16.37
20	5.89	8.79	13.51	not working
25	5.98	9.06	12.85	not working
30	6.04	9.28	12.66	not working
35	6.09	9.48	13.32	not working
40	6.14	9.67	14.40	not working

## Data Availability

The original contributions presented in the study are included in the article. Further inquiries can be directed to the corresponding author.
